# Intestinal Parasite Profile in the Stool of HIV Positive Patients in relation to Immune Status and Comparison of Various Diagnostic Techniques with Special Reference to* Cryptosporidium* at a Tertiary Care Hospital in South India

**DOI:** 10.1155/2016/3564359

**Published:** 2016-07-17

**Authors:** Vishnu Kaniyarakkal, Nizamuddin Mundangalam, Anitha Puduvail Moorkoth, Sheela Mathew

**Affiliations:** ^1^Department of Microbiology, Government Medical College Kozhikode, Kozhikode 673016, India; ^2^Department of Infectious Diseases, Government Medical College Kozhikode, Kozhikode 673016, India

## Abstract

Acquired immunodeficiency syndrome and related opportunistic infections are a significant cause of morbidity and mortality in susceptible population. This study aims to negate the paucity of data regarding the relation between CD4 levels, prevalence of enteric parasites, and the outcome of treatment with HAART (highly active antiretroviral therapy) and Cotrimoxazole in Kerala, India. Multiple stool samples from 200 patients in a cross-sectional study were subjected to microscopy and* Cryptosporidium* stool antigen ELISA. Parasites were identified in 18 samples (9%).* Cystoisospora* and* Cryptosporidium* spp. were seen in 9 cases (4.5%) and 5 cases (2.5%), respectively.* Microsporidium* spores and* Chilomastix mesnili* cysts were identified in 1 case each (0.5% each). Seven cases of* Cystoisospora* diarrhoea recovered after treatment with Cotrimoxazole. Diarrhoea due to* Cryptosporidium* spp. in all 5 cases subsided after immune reconstitution with HAART. This study concludes that a positive association was seen between low CD4 count (<200 cells/*μ*L) and overall parasite positivity (*P* value < 0.01). ELISA is a more sensitive modality for the diagnosis of* Cryptosporidium* diarrhoea.* Chilomastix mesnili*, generally considered a nonpathogen, may be a cause of diarrhoeal disease in AIDS. Immune reconstitution and Cotrimoxazole prophylaxis remain to be the best therapeutic approach in AIDS-related diarrhoea.

## 1. Introduction

Infection with human immunodeficiency virus (HIV) and its end stage, acquired immunodeficiency syndrome (AIDS), is a major public health challenge of modern times. Diarrhoea caused by parasites is one of the major opportunistic illnesses in HIV/AIDS resulting in significant morbidity. It diminishes patients' quality of life and if persistent causes dehydration, poor nutrition, and weight loss. Diarrhoea has been associated with 50% of HIV/AIDS patients in the developed world and in up to 100% of patients residing in developing countries [1]. The etiological enteric parasitic agents vary from patient to patient and from country to country depending on the geographical distribution, endemicity, seasonal variation of pathogens, and also the immune status of the patient [1].

HIV and parasitic infections may interact and mutually affect one another and parasitoses may facilitate the progression from asymptomatic HIV infection to AIDS. The common immunopathogenetic basis for the deleterious effects that parasitic diseases may have on the natural history of HIV infection involves a preferential activation of the T helper (Th2) type process. Thus the control of parasitic diseases is also necessary to aid in combating the HIV pandemic [2].

Since the start of the HIV epidemic, around 78 million people have become infected and 39 million have died of AIDS-related illnesses [3]. In 2013, of the 4.8 million people living with HIV in Asia and the Pacific, 250 000 died of AIDS-related causes [3]. India accounts for 51% of all AIDS-related deaths in the region [3].

There is paucity of data on relationship of CD4 levels and HIV/AIDS status with prevalence of enteric parasites among the HIV patients from Kerala, India. The present study therefore aims to determine the profile of enteric parasites and to study their association with immune status in HIV patients registered at the antiretroviral treatment centre of a tertiary care hospital in Kerala. Emphasis is also given on the comparison between various diagnostic techniques.

## 2. Materials and Methods

The study was conducted among 200 HIV seropositive patients registered at the antiretroviral treatment centre of Government Medical College Kozhikode, over a period of one year from January 2013 to December 2013. A clinical workup comprising history, WHO staging of the disease, antiretroviral treatment status, presence or absence of diarrhoea, Cotrimoxazole prophylaxis, and source of drinking water was constructed using a structured questionnaire. Flow cytometry (CyFlow Counter, Partec) was used to assess the CD4 T cell count and expressed as cells per cubic millimetre of blood (cells/*μ*L).

A minimum of three feces samples were obtained from each patient on separate days. Concentration was done by formol ether sedimentation technique. Microscopy of wet mount and smear preparation was carried out before and after concentration. Smears were stained by Kinyoun's acid-fast method, rapid field stain, and modified trichrome stain (Ryan Blue method) for detection of trophozoites and cyst forms of parasites including the spores of* Microsporidia*.* Cryptosporidium* antigen stool ELISA (DRG Instruments GmbH, Frauenbergstr. 18, 35039 Marburg) was performed on samples with CD4 T cell count <200. Bacterial and fungal culture was done on all samples to rule out nonparasitic infectious causes of diarrhoea.

## 3. Results

Of the 200 HIV positive stool samples studied, 136 (68%) were of males and 64 (32%) were of females. 91 patients (45.5%) had acute or chronic diarrhoea and 109 (54.5%) patients did not have diarrhoea. 147 patients (73.5%) included in the study were on ART and 53 patients (26.5%) were ART naive. 58 (29%) were on Cotrimoxazole prophylaxis and the rest of the subjects were not on Cotrimoxazole prophylaxis. 37 subjects (74%) with severe immunosuppression (CD4 count <200) presented with diarrhoea and 26 subjects (34.2%) with no immunosuppression (CD4 count >500) presented with diarrhoea (Table 1).

**Figure 1:**
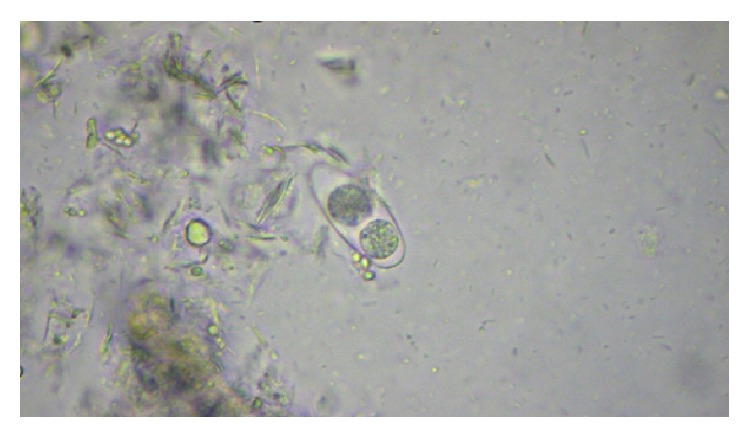
Mature* Cystoisospora* oocyst on normal saline wet mount, under high power.

**Figure 2 fig2:**
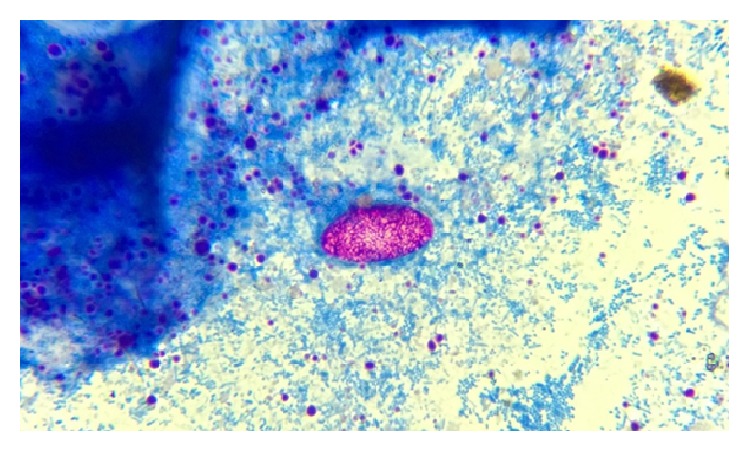
Immature* Cystoisospora* oocyst on modified acid-fast stain, under oil immersion.

**Figure 3:**
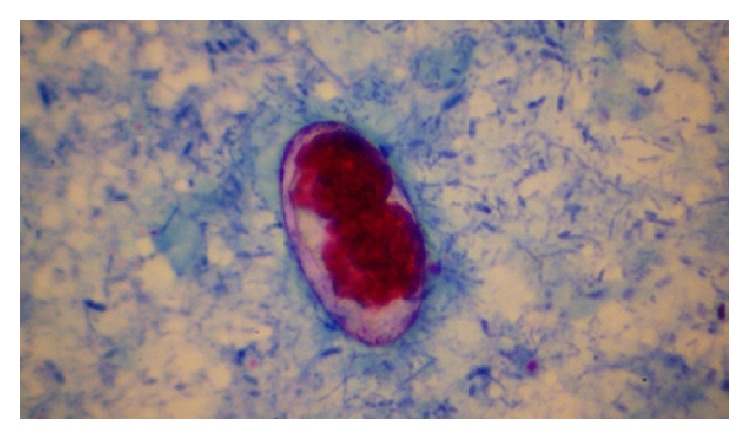
Mature* Cystoisospora* oocyst with two sporoblasts on modified acid-fast stain, under oil immersion.

**Figure 4:**
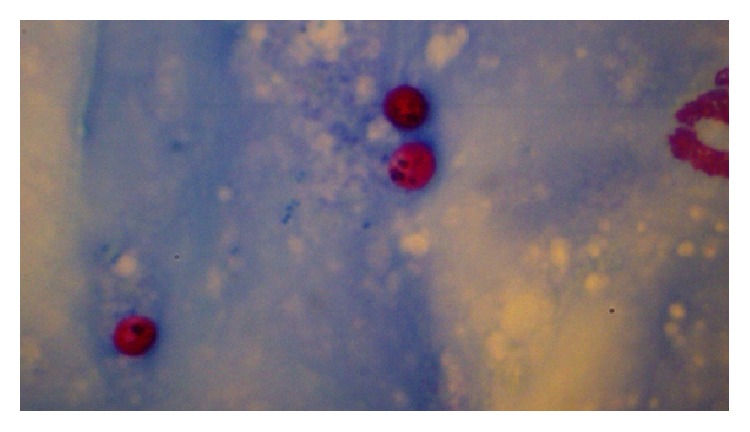
*Cryptosporidium* oocysts on modified acid-fast stain, under oil immersion.

**Figure 5:**
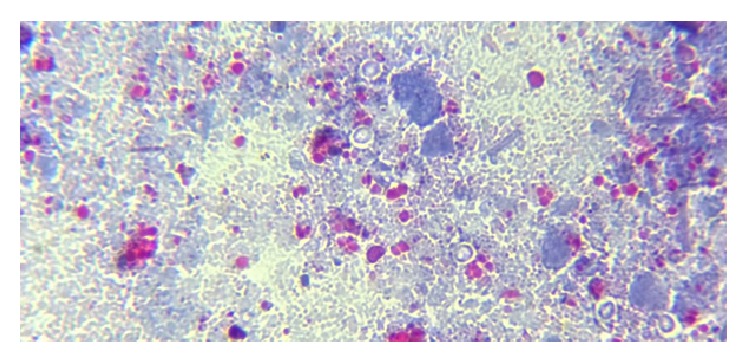
*Cryptosporidium* oocysts appearing as unstained structures on modified trichrome stain, under oil immersion.

**Figure 6:**
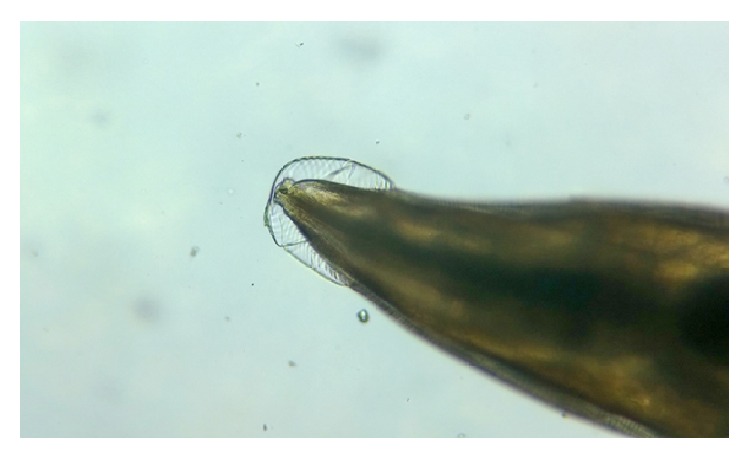
Cervical alae of* Enterobius vermicularis*, under low power.

**Figure 7:**
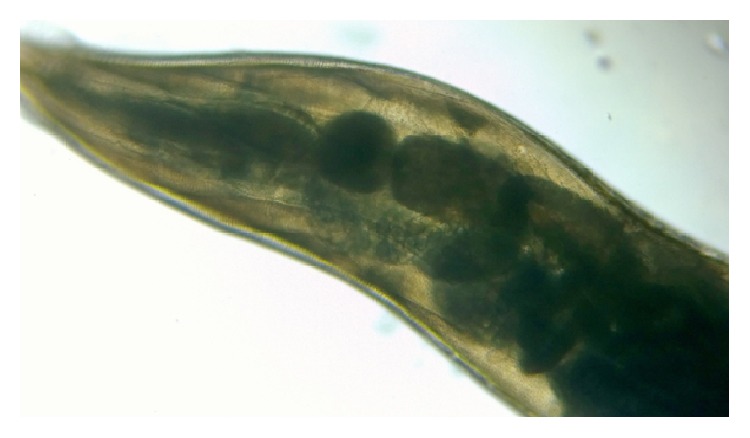
Double bulb oesophagus of* Enterobius vermicularis*, under low power.

**Figure 8:**
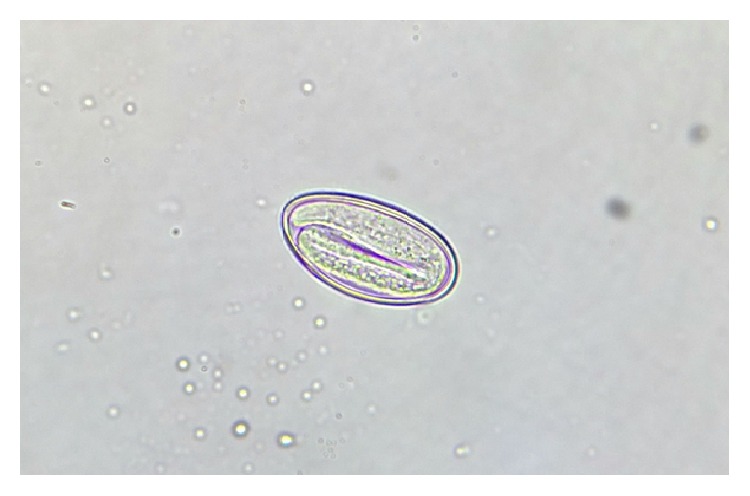
Non-bile-stained ovum of* Enterobius vermicularis* with larva inside on normal saline wet mount, under high power.

**Figure 9:**
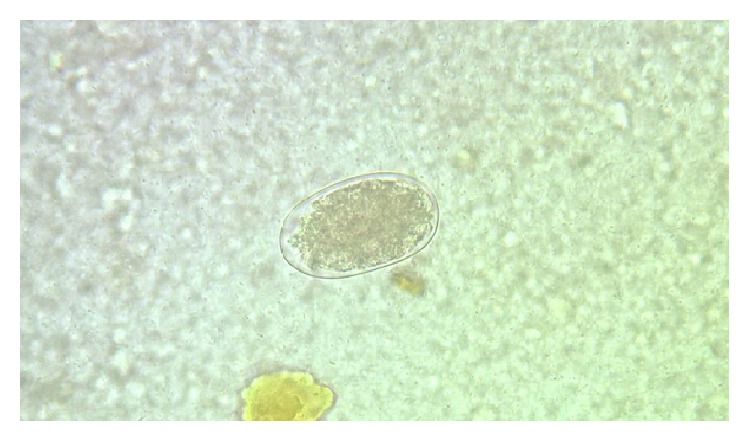
Non-bile-stained ovum of hookworm with segmented embryo inside on normal saline wet mount, under high power.

**Figure 10:**
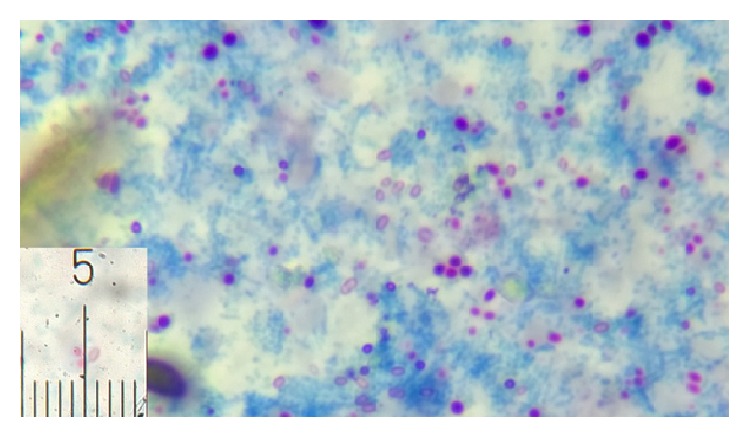
Microsporidial spores stained reddish pink with horizontal stripe measuring 1.9 *μ*m (inset) on modified acid-fast stain, under oil immersion.

**Figure 11:**
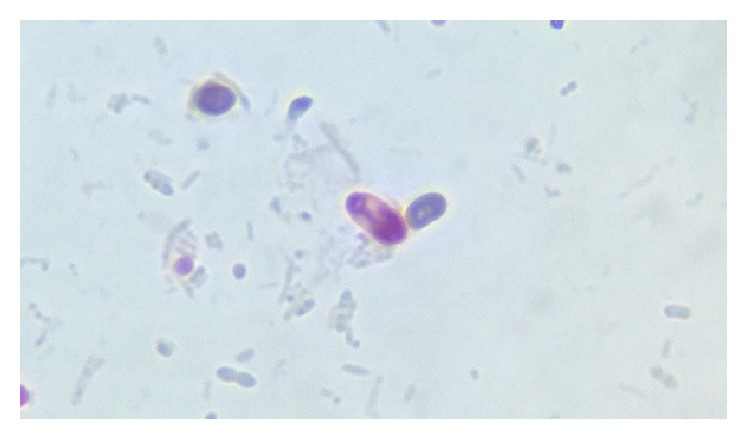
Magnified view of microsporidial spore on modified trichrome stain, under oil immersion.

**Figure 12:**
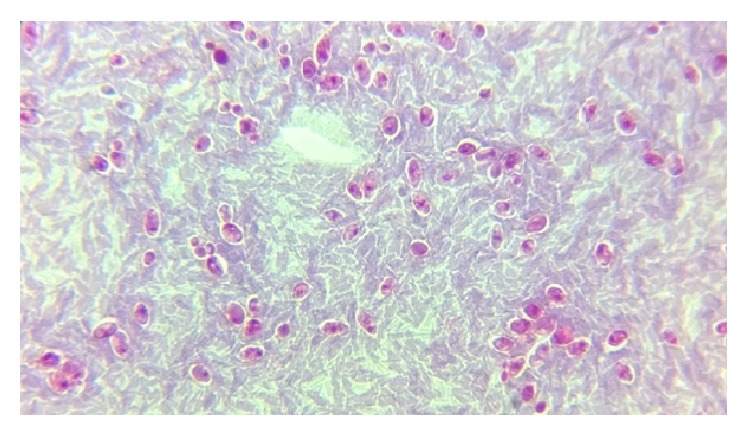
Cysts of* Chilomastix mesnili* on modified trichrome stain, under oil immersion.

**Figure 13:**
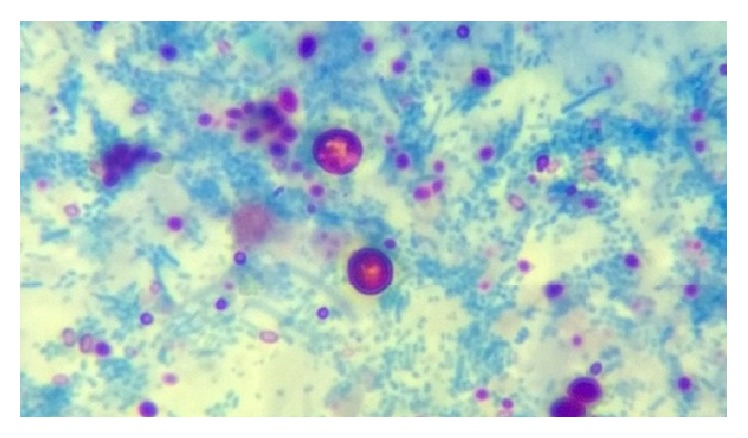
Variably acid-fast* Cryptosporidium* oocysts and microsporidial spores on modified acid-fast stain, under oil immersion.

Parasites were identified in 18 samples (9%).* Cystoisospora* oocysts (Figures 1 [Fig fig2]–3) were identified in 9 cases (4.5%) and* Cryptosporidium* oocysts (Figures 4 and 5) in 5 cases (2.5%).* Enterobius vermicularis* worm (Figures 6 and 7) and ova (Figure 8) were identified in 2 cases (1%) and hookworm ova (Figure 9),* Microsporidium* spores (Figures 10 and 11), and* Chilomastix mesnili* cysts (Figure 12) in 1 case each (0.5% each). One subject had mixed infection with both* Cryptosporidium* spp. and* Microsporidium* spp. (Figure 13). This study shows a positive association of low CD4 count with diarrhoea and parasite positivity (both with *P* value < 0.01). Also majority of the parasite positive cases (83.3%) presented with diarrhoea (*P* value = 0.001).

All* Cryptosporidium* oocyst positive cases in this study were seen in subjects with a CD4 cell count <200 (severe immunosuppression). Thus there is a positive association between* Cryptosporidium* positivity in stool and a CD4 cell count of <200 (*P* value = 0.002). On the other hand, there is no significant association between CD4 cell count <200 (severe immunosuppression) and* Cystoisospora* positivity in this study (*P* value of 0.06).

Only 2 cases of* Cryptosporidium* diarrhoea were diagnosed by modified acid-fast staining of stool samples, whereas 5 cases were diagnosed to have* Cryptosporidium* diarrhoea by stool antigen ELISA (Table 2). The study shows that stool ELISA is a better diagnostic modality than stool modified acid-fast stain for the diagnosis of* Cryptosporidium* (*P* value < 0.01). The 9 cases of* Cystoisospora* infection in this study were demonstrated by both wet mount and modified acid-fast stain. In all the 9 cases,* Cystoisospora* oocysts were demonstrated in modified acid-fast stain on primary examination itself. But in only 5 cases, oocysts were demonstrated at preliminary examination by wet mount, the other 4 being demonstrated in wet mount on retrospective examination. The* Cystoisospora* oocysts in these 5 cases, 2 cases of* Enterobius vermicularis,* and 1 case of hook worm were the only parasites demonstrated on wet mount before concentration. In all the other cases, parasites were demonstrated either on wet mount or by staining of concentrated samples only.

One of the* Cryptosporidium* positive cases and 6 of the* Cystoisospora* positive cases (38.9%) were identified on repeated stool sample examination only. One case positive for* Cystoisospora* was identified on examination of the sixth stool sample.

Of the five patients diagnosed to have* Cryptosporidium* infection, diarrhoea subsided in four, after a change of ART regimen from ZLN (Zidovudine, Lamivudine, and Nevirapine) to TLE (Tenofovir, Lamivudine, and Efavirenz). Fifth patient had complete remission of symptoms after he was started on first-line ART. No other treatment specific for* Cryptosporidium* was instituted in these patients.

Two patients out of nine who were diagnosed with* Cystoisospora* infection succumbed to the disease and expired. Out of these two patients, Cotrimoxazole could not be given to one because of hypersensitivity reactions and the other continued to have diarrhoea despite therapy with Cotrimoxazole. The rest of the seven patients became symptom-free after they were started on Cotrimoxazole prophylaxis as per National AIDS Control Organisation guidelines.

In this study bacterial culture for* Salmonella*,* Shigella*, and* Vibrio cholerae* yielded no pathogen. Fungal culture was also negative for opportunistic fungi causing diarrhoea.

## 4. Discussion

In Asia, the highest numbers of HIV-infected individuals belong to India and China [4]. The most common parasites causing diarrhoea in HIV-infected individuals include* Cryptosporidium parvum*,* Isospora belli*,* Microsporidium* spp.,* Giardia intestinalis*,* Entamoeba histolytica,* and* Strongyloides stercoralis* [4]. This study determined the profile of intestinal parasites among HIV positive individuals and attempted to investigate whether the distribution of parasites was affected by immune status. Also, different diagnostic techniques were compared to determine the more effective and practical one to be used in resource poor settings.

Majority of the subjects in the study were males (68%). Studies from other parts of India also show a higher proportion of males among HIV-infected population (61%–64%) [5, 6]. This male preponderance should be because of the propensity of males to travel outside hometown for work and greater exposure to promiscuous and unprotected sex. Diarrhoea was seen in 45.5% of the subjects which is consistent with the data available from developing countries [7–9]. Overall parasite positivity in the study was 9%. The prevalence of intestinal parasitic infections in HIV-infected patients from developing countries ranges from 12% to 38% [9–12]. The lower prevalence of parasites reported in this study could be due to the fact that stool examinations were performed whether or not the patients had diarrhoea. In this study, among patients with diarrhoea, the parasite positivity was 16%.

*Cystoisospora* was identified in maximum number of cases followed by* Cryptosporidium* in this study. In various studies conducted in north India and other countries, the most common parasite was* Cryptosporidium* [13, 14]. But studies from south India had findings similar to the one in this study [15, 16]. This difference may be attributed to the variation in geographical habitat of parasites and climate. Mixed infection with* Cryptosporidium* and* Microsporidium* was seen in one patient identified by both modified acid-fast staining and modified trichrome staining. Studies substantiate the fact that mixed infection with* Cryptosporidium* spp. and* Microsporidium* spp. is indeed common among HIV positive population [17–19]. Positive association between a CD4 count of <200 (severe immunosuppression) and parasite positivity in general was seen in this study (*P* value < 0.01). This association was not seen in case of* Cystoisospora* positive cases. These findings are corroborated in studies conducted worldwide [9, 14, 20, 21].* Chilomastix mesnili* cysts were identified in one patient with CD4 count <200 cells/*μ*L, showing the pathogenic nature of this otherwise nonpathogenic parasite in HIV patients [22]. The nature of periodic shedding of parasites necessitates multiple stool sample examinations for accurate diagnosis [23].

Immune reconstitution with HAART is the best therapeutic approach in diarrhoea due to* Cryptosporidium.* Even though treatment with Cotrimoxazole is effective for diarrhoea caused by* Cystoisospora*, a possibility of Cotrimoxazole resistant cystoisosporiasis should be borne in mind [24, 25].

This study shows a positive association between consumption of tap water and parasite positivity (*P* value = 0.004). Two subjects consuming boiled water at home also were diagnosed with parasitosis. This can be attributed to the unreliability of quality of water and food that one is exposed to, while travelling in a developing country like India. In a study conducted in Nepal, although a statistically significant association between the source of drinking water and parasite positivity was not seen, 20% of those taking direct tap water for drinking purposes and 12.5% of those using bore well water had intestinal parasitosis [10].

## 5. Conclusions

This study underscores the importance of routine screening for intestinal parasites in the stool of HIV patients with severe immunosuppression and diarrhoeal symptoms. Diarrhoea due to* Cystoisospora* is more common in south Indian settings. Mixed infections with* Cryptosporidium* and* Microsporidium* are not uncommon, necessitating a high index of suspicion and the use of different staining methods. While there was a positive association between severe immunosuppression and* Cryptosporidium* positivity, no such association was seen in case of cystoisosporiasis. ELISA is a better modality for the diagnosis of Cryptosporidial diarrhoea and should be included in the diagnostic depository where possible.* Chilomastix mesnili*, generally considered a nonpathogen, may be a cause of diarrhoeal disease in HIV positive population. The association of* Cystoisospora* infection with mortality necessitates the prompt institution of Cotrimoxazole prophylaxis and effective supportive therapy. In diarrhoea due to* Cryptosporidium*, treatment should always be aimed at immune reconstitution.

## Figures and Tables

**Table 1:** Results.

Patient characteristics	All patients (*n* = 200)	Patients with intestinal parasites (*n* = 18)	Patients without intestinal parasites (*n* = 182)	*P* value
Gender male, *n* (%)	136 (68%)	17 (94.4%)	119 (65.4%)	0.012
WHO staging of HIV, *n* (%)				0.076
Stage 1	11 (5.5%)	0 (0%)	11 (6%)	
Stage 2	34 (17%)	2 (11%)	32 (17.6%)	
Stage 3	145 (72.5%)	13 (72.2%)	132 (72.5%)	
Stage 4	10 (5%)	3 (16.7%)	7 (3%)	
Diarrhoea, *n* (%)	91 (45.5%)	15 (83.3%)	76 (41.8%)	0.001
Immune status, *n* (%)				0.000
No immunosuppression (CD4 > 500 cells/*µ*L)	76 (38%)	1 (5.6%)	75 (41.2%)	
Mild immunosuppression (CD4 350–499 cells/*µ*L)	40 (20%)	3 (16.7%)	37 (20.3%)	
Advanced immunosuppression (CD4 200–349 cells/*µ*L)	34 (17%)	2 (11.1%)	32 (17.6%)	
Severe immunosuppression (CD4 < 200 cells/*µ*L)	50 (25%)	12 (66.7%)	38 (20.9%)	
HAART, *n* (%)	147 (73.5%)	13 (72.2%)	134 (73.6%)	0.898
Cotrimoxazole prophylaxis, *n* (%)	57 (28.5%)	5 (27.8%)	52 (28.6%)	0.943
Drinking water, *n* (%)				0.004
Boiled water	87 (43.5%)	2 (11.1%)	85 (46.7%)	
Tap water	113 (56.5%)	16 (88.9%)	97 (53.3%)	

WHO, World Health Organisation; HAART, highly active antiretroviral therapy.

**Table 2:** *Cryptosporidium* positive cases.

Age & sex	*Cryptosporidium *stool antigen ELISA positivity	Modified acid-fast positivity	CD4 count (cells/*µ*L)
35/male	Yes	No	40
54/male	Yes	Yes	17
38/male	Yes	No	132
38/male	Yes	No	32
69/male^*∗*^	Yes	Yes	54

^*∗*^This case presented as mixed infection along with *Microsporidium *spp.
